# Analytical solutions for two-dimensional Stokes flow singularities in a no-slip wedge of arbitrary angle

**DOI:** 10.1098/rspa.2017.0134

**Published:** 2017-06-07

**Authors:** Darren G. Crowdy, Samuel J. Brzezicki

**Affiliations:** Department of Mathematics, Imperial College London, 180 Queen’s Gate, London SW7 2AZ, UK

**Keywords:** Stokes flow, wedge, stresslet

## Abstract

An analytical method to find the flow generated by the basic singularities of Stokes flow in a wedge of arbitrary angle is presented. Specifically, we solve a biharmonic equation for the stream function of the flow generated by a point stresslet singularity and satisfying no-slip boundary conditions on the two walls of the wedge. The method, which is readily adapted to any other singularity type, takes full account of any transcendental singularities arising at the corner of the wedge. The approach is also applicable to problems of plane strain/stress of an elastic solid where the biharmonic equation also governs the Airy stress function.

## 1. Introduction

There is a well-known mathematical analogy between the slow motion of a viscous fluid in two dimensions, and systems of plane stress and strain of a linear elastic solid [1]. Both systems are governed by a biharmonic field equation: in low-Reynolds-number fluid dynamics the stream function for incompressible flow of a viscous fluid is biharmonic; in plane elasticity, the relevant biharmonic field is the so-called Airy stress function. A variety of mathematical techniques have been developed to solve the biharmonic equation in planar wedge regions. In an authoritative review article on the two-dimensional biharmonic equation, Meleshko [2] discusses solving it in wedges and gives an interesting history of the problem. There is a huge literature, especially in plane elasticity, on biharmonic problems forced by various boundary loadings, including at the apex of the wedge, and the reader is referred to [2] more references.

The focus of this article is, however, on problems in a wedge driven by *internal* isolated singularities. Moreover, we focus on the mathematical problem as it appears in modelling slow viscous Stokes flows.

It is known that, at the corner of a wedge, the local radial behaviour (from the apex of the wedge) of solutions to the biharmonic equations can have exponents that satisfy transcendental eigenrelations. This property of solutions of the biharmonic equation near the corner of a wedge was discovered in the plane elasticity context by Brahtz [3]. In fluid dynamics, for wedge angles less than approximately 146°, it is known that these corner singularities are responsible for the occurrence of what are now called *Moffatt eddies* [4]; in a no-slip corner these are an infinite sequence of recirculating corner eddies that get smaller, and less intense, as one descends into the corner. Dean & Montagnon [5] also studied the local nature of solutions near the corner and recognized the critical opening angle for which the exponents governing the local radial behaviour of the solutions become complex.

From a local analysis of the biharmonic equation for Stokes flows with given boundary conditions on the walls one can infer the local structure of solutions near the corner and discern their functional form [4,5]. Any corner flow will comprise a linear combination of this infinite set of local solutions. Finding which linear combination requires global information on the nature of the flow and this can be a challenging matter that is a focus of this paper. Global solutions in certain situations have nevertheless been found using special transform techniques [6].

In numerical boundary integral formulations of Stokes flow [7], as well as in simple modelling situations [8,9], the fundamental point singularities of Stokes flows are useful. A local point force singularity is known as a Stokeslet [7,10] with the next order force singularity known as the stresslet, or force dipole; the latter is commonly used in point singularity models of low-Reynolds-number organisms as they exert no net force on the fluid [9]. The stresslet can be viewed as a limit of two equal and opposite Stokeslets of increasing strength merging together (or, equivalently, it can be derived as a parametric derivative of the point Stokeslet solution with respect to the singularity location). Other irrotational singularities, sometimes called source singularities, such as the rotlet or doublet can also be considered. Jeong & Moffatt [8] used the source doublet to model the effect on a free surface of submerged counter-rotating rollers in a viscous fluid bath.

The following is a basic theoretical question: what is the flow generated by the point singularities of Stokes flow internal to a simple two-dimensional wedge geometry of arbitrary opening angle *θ* assuming, say, that the two boundaries of the wedge are no-slip walls?

We have been unable to find a general answer to this question in the literature. There are, however, isolated results for special cases. Venske [11] is apparently the first to consider the problem of a Stokeslet in a two-dimensional wedge of angle *απ*; he employed Mellin transforms but only wrote explicit expressions for solutions for the cases *α*=1 and 2. Pozrikidis [7] reports the solution for a Stokeslet in a channel geometry which can be viewed as a limit of the case *θ*→0; the construction of the solution relies on Fourier transform techniques. Crowdy & Davis [12] have solved for a point stresslet, as well as a source quadrupole, in a channel using a novel transform technique which is related to, but differs from, standard Fourier transform methods. Motivated by interest in modelling low-Reynolds-number swimming organisms, Obuse & Thiffeault [13] adapted a complex analysis approach (expounded originally by Crowdy & Samson [14] in the context of point singularities near a gap in a wall) to find the solutions for a point stresslet and a source quadrupole near a semi-infinite wall corresponding to a wedge with opening angle *θ*=2*π*. Davis & Crowdy [15] employed classical Mellin transform techniques to solve the same *θ*=2*π* problem, as well as the problem in a right-angled corner, i.e. the case *θ*=*π*/2. Those authors also briefly indicated how their Mellin transform approach could be extended to other wedge corner angles of the form *π*/*N* for *N*≥2.

The present paper can be viewed as a generalization of the analysis of Davis & Crowdy [15] to the case of *arbitrary* values of the wedge opening angle *θ* with 0<*θ*≤2*π*. Following those authors, we focus here on the case of a stresslet singularity in a wedge, not least because it allows us a direct point of comparison with previous work in the special case *θ*=*π*/2. The mathematical construction here is different to that used in [15]; it relies on a transform technique which is a generalization of that used by Crowdy & Davis [12]. The approach is readily generalized to other singularity types, including the Stokeslet singularity from which the flow due to a stresslet can alternatively be derived by taking parametric derivatives with respect to the singularity location. As a result, the new method provides a route to finding analytical solutions for any choice of Stokes flow singularity situated in any wedge geometry.

Our focus here is on the two-dimensional situation and in solving the relevant boundary-value problems completely, but we note that there are various results for a three-dimensional Stokeslet in a channel [16] and in a wedge or corner region of arbitrary angle [17,18]. We have not, however, found the general solution to the analogous two-dimensional problems documented elsewhere in the literature.

Given the mathematical analogy between slow viscous flows and plane elasticity [1] the construction here should be of value in solving boundary-value problems arising in the latter application area too [19].

## 2. Complex variable formulation of Stokes flow

With inertia ignored, at each instant in time the stream function *ψ* associated with an incompressible two-dimensional Stokes flow of a fluid of viscosity *ν* satisfies the biharmonic equation

2.1 $$\nabla^{4}\psi =0,$$

where the fluid velocity components are then (*u*,*v*)=(∂*ψ*/∂*y*,−∂*ψ*/∂*x*) and ∇^2^ is the two-dimensional Laplacian operator. On introducing the complex variable *z*=*x*+*iy* the general solution to (2.1) can be represented [20] by

2.2 ψ=Im[z¯f(z)+g(z)],

where *f*(*z*) and *g*(*z*) are two *Goursat functions* which are generally analytic in the fluid region. If *p* denotes the fluid pressure then it can be shown [20] that

2.3 $$\frac{p}{\nu }-i\omega =4f^{\prime }(z),\;u+iv=-f(z)+z\bar{f^{\prime }(z)}+\bar{g^{\prime }(z)},$$

where *ω*=−∇^2^*ψ* is the fluid vorticity. The prime notation is used to express derivatives with respect to the argument of the function.

At regular points in the flow the Goursat functions *f*(*z*) and *g*(*z*) are analytic functions but they can also possess isolated singularities that can be used to model particular physical scenarios. The most fundamental singularity is the Stokeslet, which corresponds to an isolated point force in the flow [7,10,20]. The point stresslet is also a well-known basic singularity in the theory of slow viscous flows [7,10,20] and it is this singularity type that we will focus on here mainly because recent activity in modelling the locomotion of force- and torque-free micro-organisms in low-Reynolds-number flows makes it of special interest and because, as mentioned above, the problem for a stresslet in a wedge with a right-angled corner, *θ*=*π*/2, has recently been solved using different methods [15] thereby affording us the opportunity of a direct check on our independent method of solution.

The form of the singularities of the Goursat functions associated with a torque-free stresslet at some complex-valued position *z*_0_ was written down by Crowdy & Or [9] and has the local form

2.4 $$\begin{array}{rl} f(z) & =\frac{\mu }{z-z_{0}}+\text{locally analytic function},\\ g(z) & =-\frac{\mu \bar{z_{0}}}{\left(z-z_{0}\right)}+\text{locally analytic function}, \end{array}\}$$

where μ∈C is some complex-valued constant (the ‘stresslet strength’). If the stresslet is the only singularity in the flow then *f*(*z*) and *g*(*z*) must be analytic everywhere else in the fluid domain. In a wedge with solid walls these functions must also be such that the no-slip conditions are satisfied on the two walls.

We will find an analytical expression for the flow in a wedge of arbitrary opening angle *θ* due to such a stresslet singularity located at some point *z*_0_ interior to the wedge. In §7, we confirm that the solution retrieves results known for special cases of *θ* already reported in the literature.

## 3. Conformal mapping to a channel region

While boundary-value problems for the biharmonic equation are not generally conformally invariant, the key to the success of the analysis here is the use of a conformal mapping to transplant the wedge region in a complex *z*-plane to a channel region in a complex parametric *η*-plane. Consider a stresslet in a wedge of angle *θ*. Let

3.1 $$\eta =\log z,\;z=e^{\eta }.$$

This conformal mapping transplants the corner region 0<*arg*[*z*]<*θ* to an infinite strip in the *η*-plane with

3.2 −∞<Re[η]<∞and0<Im[η]<θ.

The stresslet at *z*_0_ will be transplanted to a singularity at *η*_0_ where

3.3 $$\eta_{0}=\log z_{0},\;z_{0}=e^{\eta_{0}}.$$

**Figure 1.**
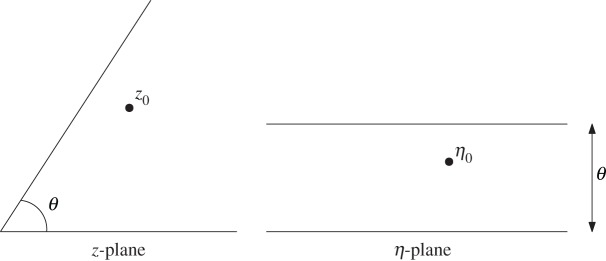
Conformal mapping (3.1) from the wedge region in the *z*-plane to a channel in a parametric *η*-plane.

A schematic of the transformation is given in figure 1.

It will turn out to be more convenient to solve for the required analytic functions in the *η*-channel than in the physical wedge region of the *z*-plane and this observation is essential to our approach. It is important to emphasize, however, that owing to the lack of conformal invariance of the boundary-value problem, after the wedge domain is mapped conformally to a channel the boundary-value problem to be solved in that new channel geometry does *not* correspond to that for a point stresslet in a no-slip channel as already solved by Crowdy & Davis [12].

## 4. Mathematical preliminaries

It is worth recording some mathematical preliminaries. Since, by a Taylor expansion,

4.1 $$\begin{array}{rl} z-z_{0} & =z^{\prime }(\eta_{0})(\eta -\eta_{0})+\frac{z^{\prime \prime }(\eta_{0})}{2!}{\left(\eta -\eta_{0}\right)}^{2}+\frac{z^{\prime \prime \prime }(\eta_{0})}{3!}{\left(\eta -\eta_{0}\right)}^{3}+\cdots \\ & =z_{0}(\eta -\eta_{0})\left[1+\frac{\eta -\eta_{0}}{2}+\frac{{\left(\eta -\eta_{0}\right)}^{2}}{6}+\cdots \right] \end{array}$$

then, near *η*=*η*_0_, we can write

4.2 $$\begin{array}{rl} \frac{1}{\eta -\eta_{0}} & =\frac{z_{0}}{z-z_{0}}\left[1+\frac{\eta -\eta_{0}}{2}+\frac{{\left(\eta -\eta_{0}\right)}^{2}}{6}+\cdots \right]\\ & =\frac{z_{0}}{z-z_{0}}+\frac{z_{0}}{2}\left[\frac{\eta -\eta_{0}}{z-z_{0}}\right]+\frac{z_{0}}{6}\left[\frac{\eta -\eta_{0}}{z-z_{0}}\right](\eta -\eta_{0})+\cdots . \end{array}$$

Now

4.3 $$\begin{array}{rl} \eta -\eta_{0} & =\eta^{\prime }(z_{0})(z-z_{0})+\frac{\eta^{\prime \prime }(z_{0})}{2!}{\left(z-z_{0}\right)}^{2}+\frac{\eta^{\prime \prime \prime }(z_{0})}{3!}{\left(z-z_{0}\right)}^{3}+\cdots \\ & =\frac{1}{z_{0}}(z-z_{0})-\frac{1}{2z_{0}^{2}}{\left(z-z_{0}\right)}^{2}+\frac{1}{3z_{0}^{3}}{\left(z-z_{0}\right)}^{3}+\cdots , \end{array}$$

so that

4.4 $$\frac{\eta -\eta_{0}}{z-z_{0}}=\frac{1}{z_{0}}-\frac{1}{2z_{0}^{2}}(z-z_{0})+\frac{1}{3z_{0}^{3}}{\left(z-z_{0}\right)}^{2}+\cdots .$$

Substitution of (4.4) into (4.2) gives

4.5 $$\frac{1}{\eta -\eta_{0}}=\frac{z_{0}}{z-z_{0}}+\frac{1}{2}-\frac{1}{12z_{0}}(z-z_{0})+\frac{1}{24z_{0}^{2}}{\left(z-z_{0}\right)}^{2}+\cdots .$$

This expansion will be useful later. In particular, it can be used to show that

4.6 $$\begin{array}{rl} \frac{1}{{\left(\eta -\eta_{0}\right)}^{2}} & =\frac{z_{0}^{2}}{{\left(z-z_{0}\right)}^{2}}+\frac{z_{0}}{\left(z-z_{0}\right)}+\frac{1}{12}+O{\left(z-z_{0}\right)}^{2}\\ \;\mathrm{and}\;\frac{1}{{\left(\eta -\eta_{0}\right)}^{3}} & =\frac{z_{0}^{3}}{{\left(z-z_{0}\right)}^{3}}+\frac{3z_{0}^{2}}{2{\left(z-z_{0}\right)}^{2}}+\frac{z_{0}}{2}\frac{1}{\left(z-z_{0}\right)}+O(z-z_{0}). \end{array}\}$$

To facilitate the analysis we will make use of the following 2*θi*-periodic hyperbolic functions which, near *η*_0_, have the local behaviour

4.7 $$\begin{array}{rl} & \frac{\pi }{2\theta }\coth\left(\frac{\pi (\eta -\eta_{0})}{2\theta }\right)=\frac{1}{\eta -\eta_{0}}+I_{1}(\eta ;\eta_{0},\theta ),\\ & {\left[\frac{\pi }{2\theta }\right]}^{2}\;{\mathrm{cosech}}^{2}\left(\frac{\pi (\eta -\eta_{0})}{2\theta }\right)=\frac{1}{{\left(\eta -\eta_{0}\right)}^{2}}+I_{2}(\eta ;\eta_{0},\theta )\\ \;\mathrm{and}\; & {\left[\frac{\pi }{2\theta }\right]}^{3}{\mathrm{cosech}}^{2}\left(\frac{\pi (\eta -\eta_{0})}{2\theta }\right)\coth\left(\frac{\pi (\eta -\eta_{0})}{2\theta }\right)=\frac{1}{{\left(\eta -\eta_{0}\right)}^{3}}+I_{3}(\eta ;\eta_{0},\theta ), \end{array}\}$$

where *I*_1_(*η*;*η*_0_,*θ*),*I*_2_(*η*;*η*_0_,*θ*) and *I*_3_(*η*;*η*_0_,*θ*) are functions that are analytic at *η*_0_.

## 5. A stresslet in a wedge

For a point stresslet in a wedge the Goursat functions have the local form given in (2.4) near the singularity. Define the composed functions

5.1 $$F(\eta )\equiv f(z(\eta ))\;\mathrm{and}\;G(\eta )\equiv g^{\prime }(z(\eta )).$$

Suppose

5.2 $$F(\eta )=\frac{c_{1}}{\eta -\eta_{0}},$$

for some constant *c*_1_ then, near *z*_0_,

5.3 $$f(z)=c_{1}\left[\frac{z_{0}}{z-z_{0}}+\frac{1}{2}+\cdots \right]+\text{locally analytic function},$$

so that, to be consistent with (2.4), we must choose

5.4 $$c_{1}=\frac{\mu }{z_{0}}.$$

Suppose also that

5.5 $$G(\eta )=\frac{c_{2}}{{\left(\eta -\eta_{0}\right)}^{2}}+\frac{c_{3}}{\eta -\eta_{0}}$$

for some constants *c*_2_ and *c*_3_ then, near *z*_0_,

5.6 $$\begin{array}{rl} g^{\prime }(z) & =c_{2}{\left[\frac{z_{0}}{z-z_{0}}+\frac{1}{2}+\cdots \right]}^{2}+c_{3}\left[\frac{z_{0}}{z-z_{0}}+\frac{1}{2}+\cdots \right]+\cdots \\ & =\frac{c_{2}z_{0}^{2}}{{\left(z-z_{0}\right)}^{2}}+\frac{(c_{2}+c_{3})z_{0}}{z-z_{0}}+\text{locally analytic function}, \end{array}$$

so, to be consistent with (2.4), we must ensure that

5.7 $$c_{2}=\frac{\mu \bar{z_{0}}}{z_{0}^{2}}=-c_{3}.$$

The next step is to introduce the functional decompositions

5.8 $$F(\eta )=F_{s}(\eta )+\hat{F}(\eta )\;\mathrm{and}\;G(\eta )=G_{s}(\eta )+\hat{G}(\eta ),$$

where $\hat{F}(\eta )$ and $\hat{G}(\eta )$ are taken to be analytic in the *η*-channel and decaying as |η|→∞, and

5.9 $$\begin{array}{rl} F_{s}(\eta ) & \equiv \frac{\mu }{z_{0}}\left\{\frac{\pi }{2\theta }\coth\left(\frac{\pi (\eta -\eta_{0})}{2\theta }\right)\right\}\\ G_{s}(\eta ) & \equiv \frac{\mu \bar{z_{0}}}{z_{0}^{2}}\left\{{\left[\frac{\pi }{2\theta }\right]}^{2}{\mathrm{cosech}}^{2}\left(\frac{\pi (\eta -\eta_{0})}{2\theta }\right)\right\}-\frac{\mu \bar{z_{0}}}{z_{0}^{2}}\left\{\frac{\pi }{2\theta }\coth\left(\frac{\pi (\eta -\eta_{0})}{2\theta }\right)\right\}\\ & \;+\frac{\bar{\mu }}{\bar{z_{0}}}\left\{\frac{\pi }{2\theta }\coth\left(\frac{\pi (\eta -\bar{\eta_{0}})}{2\theta }\right)\right\}+\frac{\mu \bar{z_{0}}}{z_{0}^{2}}\left\{\frac{\pi }{2\theta }\coth\left(\frac{\pi (\eta -\bar{\eta_{0}})}{2\theta }\right)\right\}. \end{array}\}$$

The complex velocity field produced by the singular terms, i.e. the quantity

5.10 $$-\bar{F_{s}(\eta )}+\bar{z}\frac{F_{s}^{\prime }(\eta )}{z^{\prime }(\eta )}+G_{s}(\eta ),$$

vanishes as |η|→∞ owing to the inclusion of the final two terms in the expression for *G*_*s*_(*η*) which, it should be noted, are not singular at *η*_0_. It follows, from the stipulations that $\hat{F}(\eta )$ and $\hat{G}(\eta )$ also decay in the far-field, that the *total* velocity field components associated with the ansatz (5.8) will tend to zero as |η|→∞. It can be verified that *F*(*η*) and *G*(*η*) have the required singularity structure (2.4) at *z*_0_. To see this note that, as *z*→*z*_0_, *η*→*η*_0_,

5.11 $$\begin{array}{rl} F_{s}(\eta ) & =\frac{\mu }{z-z_{0}}+\frac{\mu }{z_{0}}\left[\frac{1}{2}+I_{1}(\eta_{0};\eta_{0},\theta )\right]+O(z-z_{0})\\ \;\mathrm{and}\;G_{s}(\eta ) & =\frac{\mu \bar{z_{0}}}{{\left(z-z_{0}\right)}^{2}}+\frac{\mu \bar{z_{0}}}{z_{0}^{2}}\left[-\frac{5}{12}-I_{1}(\eta_{0};\eta_{0},\theta )+I_{2}(\eta_{0};\eta_{0},\theta )\right]\\ & \;+\left[\frac{\bar{\mu }}{\bar{z_{0}}}+\frac{\mu \bar{z_{0}}}{z_{0}^{2}}\right]\frac{\pi }{2\theta }\coth\left[\frac{\pi (\eta_{0}-\bar{\eta_{0}})}{2\theta }\right]+O(z-z_{0}), \end{array}\}$$

where we have used (4.5)–(4.7).

## 6. A transform method

The task now is to find $\hat{F}(\eta )$ and $\hat{G}(\eta )$. This will be done by means of a transform technique similar in spirit to the transform approach to solving for the fundamental Stokes flow singularities in a channel recently given by Crowdy & Davis [12]; the reader is referred there for more details on the background of this method. It has close connections to the classical complex Mellin transform but our approach below, involving the statement and analysis of so-called global relations, stems from ideas associated with the unified transform method of Fokas and collaborators [21,22].

The following representation pertains for a function analytic in the *η*-strip [12]:

6.1 $$\hat{F}(\eta )=\frac{1}{2\pi }\int_{L_{1}}\rho_{1}(k)\;e^{ik\eta }dk+\frac{1}{2\pi }\int_{L_{2}}\rho_{2}(k)\;e^{ik\eta }dk,$$

where *L*_1_ is the ray, in the complex *k*-plane, from the origin along the positive real axis while *L*_2_ is the ray from the origin along the negative real axis and where the so-called *spectral functions* are defined to be

6.2 $$\begin{array}{r} \rho_{1}(k)=\int_{-\infty }^{\infty }\hat{F}(\eta )\;e^{-ik\eta }d\eta \;\mathrm{and}\;\rho_{2}(k)=\int_{i\theta +\infty }^{i\theta -\infty }\hat{F}(\eta )\;e^{-ik\eta }d\eta . \end{array}$$

These spectral functions satisfy a *global relation* [12] given by

6.3 $$\rho_{1}(k)+\rho_{2}(k)=0,\;k\in R.$$

It follows that we can write

6.4 $$\hat{F}(\eta )=\frac{1}{2\pi }\int_{-\infty }^{\infty }\rho_{1}(k)\;e^{ik\eta }dk.$$

There is an analogous representation for $\hat{G}(\eta )$; the corresponding spectral functions will be represented as *ρ*_3_(*k*) and *ρ*_4_(*k*) with

6.5 $$\rho_{3}(k)+\rho_{4}(k)=0,\;k\in R.$$

Hence,

6.6 $$\hat{G}(\eta )=\frac{1}{2\pi }\int_{-\infty }^{\infty }\rho_{3}(k)\;e^{ik\eta }\;dk.$$

To solve the problem of interest it is enough to determine *ρ*_1_(*k*) and *ρ*_3_(*k*).

The spectral functions *ρ*_1_(*k*) and *ρ*_3_(*k*) can be found by analysing the boundary conditions and the global relations (6.3) and (6.5). It can be shown that

6.7 $$\int_{-\infty }^{\infty }\bar{\hat{F}(\eta )}\;e^{-ik\eta }\;d\eta =\bar{\rho_{1}}(-k)$$

and, similarly, that

6.8 $$\int_{i\theta +\infty }^{i\theta -\infty }\bar{\hat{F}(\eta )}\;e^{-ik\eta }d\eta =\;e^{2k\theta }\bar{\rho_{2}}(-k).$$

These are established by taking the complex conjugates of *ρ*_1_(*k*) and *ρ*_2_(*k*) as defined in (6.2) and letting $\bar{k}\mapsto k$.

The no-slip boundary conditions on the two sidewalls of the wedge region are

6.9 $$-\bar{f(z)}+\bar{z}f^{\prime }(z)+g^{\prime }(z)=0,\;\arg[z]=0,\theta .$$

On substitution of (5.8), and use of the facts that $\bar{z}=z$ on the lower channel wall while $\bar{z}=e^{-2i\theta }z$ on the upper channel wall, these boundary conditions become

6.10 $$-\bar{\hat{F}(\eta )}+z\frac{d}{dz}\hat{F}(\eta )+\hat{G}(\eta )=\bar{F_{s}(\eta )}-z\frac{d}{dz}F_{s}(\eta )-G_{s}(\eta ),\;(\text{lower wall})$$

and

6.11 $$-\bar{\hat{F}(\eta )}+e^{-2i\theta }z\frac{d}{dz}\hat{F}(\eta )+\hat{G}(\eta )=\bar{F_{s}(\eta )}-e^{-2i\theta }z\frac{d}{dz}F_{s}(\eta )-G_{s}(\eta ),\;(\text{upper wall}).$$

But, from (3.1), and use of the chain rule,

6.12 $$z\frac{d}{dz}=\frac{d}{d\eta }.$$

We now multiply each of the boundary conditions (6.10) and (6.11) by the factor e^−i*kη*^ and integrate along the respective boundaries to produce

6.13 $$\begin{array}{rl} -\bar{\rho_{1}}(-k)+ik\rho_{1}(k)+\rho_{3}(k) & =R_{1}(k)\\ \;\mathrm{and}\;-e^{2k\theta }\bar{\rho_{2}}(-k)+e^{-2i\theta }ik\rho_{2}(k)+\rho_{4}(k) & =R_{2}(k), \end{array}\}$$

where

6.14 $$\begin{array}{rl} R_{1}(k) & \equiv \int_{-\infty }^{\infty }\left[\bar{F_{s}(\eta )}-\frac{d}{d\eta }F_{s}(\eta )-G_{s}(\eta )\right]e^{-ik\eta }\;d\eta \\ \;\mathrm{and}\;R_{2}(k) & \equiv \int_{i\theta +\infty }^{i\theta -\infty }\left[\bar{F_{s}(\eta )}-e^{-2i\theta }\frac{d}{d\eta }F_{s}(\eta )-G_{s}(\eta )\right]e^{-ik\eta }\;d\eta . \end{array}\}$$

The two functions *R*_1_(*k*) and *R*_2_(*k*) are computable because *F*_*s*_(*η*) and *G*_*s*_(*η*) are defined in (5.9). Addition of the two equations in (6.13) implies that

6.15 $$-\bar{\rho_{1}}(-k)-e^{2k\theta }\bar{\rho_{2}}(-k)+ik\rho_{1}(k)+e^{-2i\theta }ik\rho_{2}(k)+\rho_{3}(k)+\rho_{4}(k)=R(k),$$

where we define

6.16 $$R(k)\equiv R_{1}(k)+R_{2}(k).$$

Use of the two global relations (6.3) and (6.5) then implies that

6.17 $$(e^{2k\theta }-1)\bar{\rho_{1}}(-k)+ik(1-e^{-2i\theta })\rho_{1}(k)=R(k),\;k\in R.$$

The Schwarz conjugate equation is

6.18 $$(e^{2k\theta }-1)\rho_{1}(-k)-ik(1-e^{2i\theta })\bar{\rho_{1}}(k)=\bar{R}(k),\;k\in R.$$

Now let *k*↦−*k* in (6.18):

6.19 $$(e^{-2k\theta }-1)\rho_{1}(k)+ik(1-e^{2i\theta })\bar{\rho_{1}}(-k)=\bar{R}(-k),\;k\in R.$$

Now (6.17) implies that

6.20 $$\bar{\rho_{1}}(-k)=\frac{R(k)-ik(1-e^{-2i\theta })\rho_{1}(k)}{e^{2k\theta }-1}.$$

On substitution into (6.19) we find, after rearrangement, that

6.21 $$\rho_{1}(k)=\frac{\left(e^{2k\theta }-1)\bar{R}(-k)-ik(1-e^{2i\theta })R(k\right)}{\left(e^{2k\theta }-1)(e^{-2k\theta }-1)+k^{2}(1-e^{2i\theta })(1-e^{-2i\theta }\right)}.$$

Since *R*(*k*) is known, (6.21) gives the spectral function needed to determine $\hat{F}(\eta )$ via formula (6.4). Since *R*_1_(*k*) is also a known function, the first formula in (6.13) then gives the spectral function *ρ*_3_(*k*) needed to find $\hat{G}(\eta )$ from (6.6). In appendix A explicit formulae for *R*(*k*) and *R*_1_(*k*) are derived.

Now from (2.3) we have

6.22 $$4f^{\prime }(z)=\frac{p}{\nu }-i\omega .$$

It follows, from the chain rule, that

6.23 $$\frac{p}{\nu }-i\omega =4\frac{F_{s}^{\prime }(\eta )}{z^{\prime }(\eta )}+4\;e^{-\eta }\left[\frac{1}{2\pi }\int_{-\infty }^{\infty }ik\rho_{1}(k)\;e^{ik\eta }\;dk\right].$$

Also,

6.24 $$u-iv=-\bar{f(z)}+\bar{z}f^{\prime }(z)+g^{\prime }(z).$$

On substitution of (5.8), and the integral representations of $\hat{F}(\eta )$ and $\hat{G}(\eta )$, we find

6.25 $$\begin{array}{rl} u-iv & =-\bar{F_{s}(\eta )}+e^{\bar{\eta }}\frac{F_{s}^{\prime }(\eta )}{z^{\prime }(\eta )}+G_{s}(\eta )-\frac{1}{2\pi }\int_{-\infty }^{\infty }\bar{\rho_{1}}(k)\;e^{-ik\bar{\eta }}\;dk\\ & \;+\frac{e^{\bar{\eta }-\eta }}{2\pi }\int_{-\infty }^{\infty }ik\rho_{1}(k)\;e^{ik\eta }\;dk+\frac{1}{2\pi }\int_{-\infty }^{\infty }\rho_{3}(k)e^{ik\eta }\;dk. \end{array}$$

On substituting for *ρ*_3_(*k*) from (6.13), the final expressions for the physical quantities *p*,*u*,*v* and *ω* associated with the point stresslet are

6.26 $$\begin{array}{rl} \frac{p}{\nu }-i\omega & =4\frac{F_{s}^{\prime }(\eta )}{z^{\prime }(\eta )}+4\;e^{-\eta }\left[\frac{1}{2\pi }\int_{-\infty }^{\infty }ik\rho_{1}(k)\;e^{ik\eta }\;rdk\right]\\ \;\mathrm{and}\;u-iv & =-\bar{F_{s}(\eta )}+e^{\bar{\eta }}\frac{F_{s}^{\prime }(\eta )}{z^{\prime }(\eta )}+G_{s}(\eta )\\ & \;+\frac{1}{2\pi }\int_{-\infty }^{\infty }[R_{1}(k)\;e^{ik\eta }+ik\rho_{1}(k)[e^{\bar{\eta }-\eta }-1]\;e^{ik\eta }\\ & \;+\bar{\rho_{1}}(k)[e^{-ik\eta }-e^{-ik\bar{\eta }}]]\;dk. \end{array}\}$$

The expression (6.26) is the required transform solution for the Stokes flow generated by a point stresslet in a wedge of general angle *θ*. Given the simple exponential relationship between *η* and *z* in (3.1) this result can be rewritten in terms of *z* alone if preferred. All functions on the right-hand side of (6.26) are known explicitly which means that evaluation of *p*,*ω*,*u* and *v* requires only simple quadrature.

## 7. Verification for special opening angles

For the three opening angles *θ*=*π*/2,*π* and 2*π*, the solution for a point stresslet in a wedge can be determined by other techniques and these results provide a check on the derived expression (6.26) which we claim is valid for general *θ*.

### (a) Special case *θ*=*π*

Consider first the case *θ*=*π*. In this case it follows from (6.21) that

7.1 $$\rho_{1}(k)=\frac{\bar{R}(-k)}{e^{-2k\pi }-1},$$

where, from (A 5), we find

7.2 $$R(k)=2\pi i\mu (1+ik)\bar{z_{0}}z_{0}^{-ik-2}+2\pi k\mu z_{0}^{-ik-1}.$$

It follows that

7.3 $$\begin{array}{rl} \hat{F}(\eta ) & =\int_{-\infty }^{\infty }\left[\frac{k\bar{\mu }(z_{0}-\bar{z_{0}}){\bar{z_{0}}}^{-ik-2}-i\bar{\mu }z_{0}{\bar{z_{0}}}^{-ik-2}}{e^{-2k\pi }-1}\right]e^{ik\eta }\;dk\\ & =\underset{J_{1}}{\underbrace{-\frac{i\bar{\mu }z_{0}}{{\bar{z_{0}}}^{2}}\int_{-\infty }^{\infty }{\left(\frac{z}{\bar{z_{0}}}\right)}^{ik}\frac{dk}{e^{-2k\pi }-1}}}+\underset{J_{2}}{\underbrace{\frac{\bar{\mu }(z_{0}-\bar{z_{0}})}{{\bar{z_{0}}}^{2}}\int_{-\infty }^{\infty }{\left(\frac{z}{\bar{z_{0}}}\right)}^{ik}\frac{k\;dk}{e^{-2k\pi }-1}}}, \end{array}$$

where we have used (3.1). Consider *J*_1_ and now suppose that $|z/\bar{z_{0}}|<1$. Then we can close the contour in the lower half plane picking up all residues at *k*=−*in* for n=0,1,2,…. The result is that

7.4 $$J_{1}=(-2\pi i)\left\{-\frac{i\bar{\mu }z_{0}}{{\bar{z_{0}}}^{2}}\right\}\sum_{n=0}^{\infty }-\frac{1}{2\pi }{\left(\frac{z}{\bar{z_{0}}}\right)}^{n}=-\frac{\bar{\mu }z_{0}}{\bar{z_{0}}}\frac{1}{z-\bar{z_{0}}}.$$

Similarly, we find

7.5 $$J_{2}=(-2\pi i)\left\{\frac{\bar{\mu }(z_{0}-\bar{z_{0}})}{{\bar{z_{0}}}^{2}}\right\}\sum_{n=1}^{\infty }\frac{\left(-in\right)}{\left(-2\pi \right)}{\left(\frac{z}{\bar{z_{0}}}\right)}^{n}.$$

On use of the identity

7.6 $$\frac{1}{{\left(1-z\right)}^{2}}=1+2z+3z^{2}+\cdots ,\;|z|<1$$

we find

7.7 $$J_{2}=\frac{\bar{\mu }(z_{0}-\bar{z_{0}})}{{\bar{z_{0}}}^{2}}\left(\frac{z}{\bar{z_{0}}}\right)\frac{1}{{\left(1-z/\bar{z_{0}}\right)}^{2}}=\frac{\bar{\mu }(z_{0}-\bar{z_{0}})}{\bar{z_{0}}}\left[\frac{1}{z-\bar{z_{0}}}+\frac{\bar{z_{0}}}{{\left(z-\bar{z_{0}}\right)}^{2}}\right].$$

Hence

7.8 $$\hat{F}(\eta )=J_{1}+J_{2}=-\frac{\bar{\mu }}{\left(z-\bar{z_{0}}\right)}+\frac{\bar{\mu }(z_{0}-\bar{z_{0}})}{{\left(z-\bar{z_{0}}\right)}^{2}}.$$

Furthermore,

7.9 $$F_{s}(\eta )=\frac{\mu }{2z_{0}}\coth\left(\frac{\eta -\eta_{0}}{2}\right)=\frac{\mu }{2z_{0}}\coth[\log{\left(z/z_{0}\right)}^{1/2}].$$

But

7.10 $$\coth[\log{\left(z/z_{0}\right)}^{1/2}]=\frac{{\left(z/z_{0}\right)}^{1/2}+{\left(z_{0}/z\right)}^{1/2}}{{\left(z/z_{0}\right)}^{1/2}-{\left(z_{0}/z\right)}^{1/2}}=\frac{z+z_{0}}{z-z_{0}}.$$

Hence

7.11 $$F_{s}(\eta )=\frac{\mu }{2z_{0}}\left[\frac{z+z_{0}}{z-z_{0}}\right]=\frac{\mu }{z-z_{0}}+\mathrm{const}.$$

leading to

7.12 $$F(\eta )=\frac{\mu }{z-z_{0}}-\frac{\bar{\mu }}{\left(z-\bar{z_{0}}\right)}+\frac{\bar{\mu }(z_{0}-\bar{z_{0}})}{{\left(z-\bar{z_{0}}\right)}^{2}}+\mathrm{const}.$$

This agrees with the result found by Crowdy & Or [9] using more direct function theoretic methods (or ‘method of images’) that pertain in this special case.

### (b) Special case *θ*=2*π*

The case *θ*=2*π* can be solved in closed form—that is, without the need to perform any numerical quadrature as required for the explicit representation (6.26)—using an adaptation of a conformal mapping method expounded by Crowdy & Samson [14], who studied point singularities in the region exterior to a gap of finite length in an infinite wall (there the geometry had two such 2*π* corners, or ‘edges’); indeed, Obuse & Thiffeault [13] adapted the latter method to study Stokes flow singularities near a semi-infinite wall. For completeness, full details of this alternative approach are given in appendix B so that the reader might affirm its very different nature. It was confirmed numerically that the system (6.26) when evaluated for *θ*=2*π* gives identical results to this conformal mapping approach. We also point out that Davis & Crowdy [15] also solved this *θ*=2*π* problem using a classical Mellin transform approach and the reader may find it instructive to compare that approach to the new transform technique used here.

### (c) Special case *θ*=*π*/2

Finally, the case *θ*=*π*/2 can also be verified against the quite different formulation using the classical Mellin transform given by Davis & Crowdy [15]. It has been confirmed numerically that the system (6.26) when evaluated at *θ*=*π*/2 gives identical results to [15].

## 8. Streamline patterns

As mentioned earlier formulae (6.26) give explicit integral expressions for all the flow quantities *p*,*ω*,*u* and *v* associated with a point stresslet; all that is needed to evaluate these, given a stresslet strength and location *μ* and *z*_0_ is a simple quadrature. It should be noted that, by construction, for points *z* strictly inside the wedge, the integrands of the two infinite line integrals over k∈(−∞,∞) decay rapidly to zero as |k|→∞ so the most straightforward way to evaluate these integrals to high accuracy is to simply truncate the integration range to some finite interval [−*L*,*L*] for some *L*>0 (that need not be too large owing to the rapid exponential decay of the integrands) and use regular quadrature techniques (the trapezoidal or Simpson’s rule).

**Figure 2.**
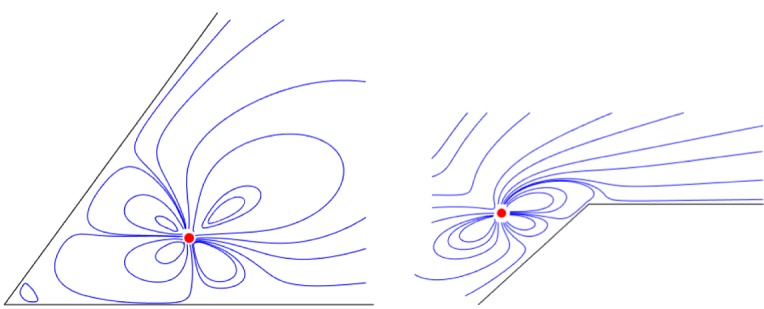
Typical streamline patterns for a stresslet of unit strength *μ*=1 with *θ*=*π*/3, *z*_0_=1+0.455*i* and *θ*=4*π*/3, *z*_0_=−0.5−0.1*i*. (Online version in colour.)

Figure 2 shows typical streamline patterns associated with a unit strength stresslet for the two choices of wedge angles *θ*=*π*/3 and *θ*=4*π*/3. The *θ*=*π*/3 opening angle is below the critical value of approximately 146° [4] for which so-called ‘Moffatt eddies’ are generated in the corner region by the stresslet.

## 9. Summary

We have derived an analytical expression (6.26) for the velocity, pressure field and vorticity field generated by a point stresslet in arbitrary position located in a no-slip wedge of arbitrary opening angle *θ*. The agreement between (6.26) with quite different solution schemes for the three special cases *θ*=*π*/2,*π* and 2*π* provides verification of the solution.

To generalize the analysis to other singularity types in a no-slip wedge requires only making the appropriate choices for the functions *F*_*s*_(*η*) and *G*_*s*_(*η*) in (5.9) and calculating the corresponding functions *R*(*k*) and *R*_1_(*k*). All subsequent steps in the analysis will be the same if the boundary conditions are that the walls of the wedge are no-slip walls. While we have focused here on no-slip walls, it is likely that the method will provide a convenient route to solution of other boundary-value problems. In that case, the relevant transform solution will follow by use of the boundary conditions and the global relations to ascertain the unknown spectral functions in the transform representation of the solution.

It is worth inspecting the denominator of the spectral function *ρ*_1_(*k*) as given in (6.21) that appears in the transform solution (6.26), namely

9.1 $$(e^{2k\theta }-1)(e^{-2k\theta }-1)+k^{2}(1-e^{2i\theta })(1-e^{-2i\theta }).$$

This can be written as

9.2 $$4(k^{2}\sin^{2}\theta -\sinh^{2}k\theta )=4(\sin^{2}\lambda \theta -\lambda^{2}\sin^{2}\theta ),\;k=i\lambda ,$$

which is precisely the eigenrelation found by Dean & Montagnon [5] and Moffatt [4] between the exponents *λ* of the radial dependence of the local solutions in the corner and the opening angle *θ*. It is by collecting residue contributions when evaluating the integrals in (6.26) using standard complex variable methods that one sees the connection between the global solution (6.26) and the local form of the corner solutions [4]. The first of an infinite sequence of eddies can be seen in the streamline plot for *θ*=*π*/3 in figure 2.

It is important to couch the present results in the context of prior work. Crowdy & Davis [12] introduced a novel transform scheme to derive the solution for a point stresslet in a channel (the case *θ*→0) and, in an appendix, those authors rederived the same solution using standard Fourier transform methods. On the other hand, Davis & Crowdy [15] used classical Mellin transform methods to solve for a point stresslet in a right-angled corner (the case *θ*=*π*/2) and the semi-infinite wall (the case *θ*=2*π*). What has been done here is to show how, by introducing a conformal mapping from the wedge to a channel in a parametric *η*-plane, the new transform method of [12] can be generalized to solve for the flow due to a point stresslet in a wedge of any angle, thereby extending the work of [15] to arbitrary angles and, furthermore, presenting a transform technique that is an alternative to the classical Mellin transform used in [15].

## Authors' contributions

Both authors contributed to the contents of this paper during the course of the second author’s PhD project under the supervision of the first author.

## Competing interests

We declare we have no competing interests.

## Funding

D.G.C. is supported from EPSRC Established Career Fellowship EP/K019430/1, a Royal Society Wolfson Merit Award and EPSRC grant no. EP/K041134/1. S.J.B. is supported by an EPSRC studentship.

## Appendix A. Calculation of *R*(*k*) and *R*_1_(*k*)

On substitution of (5.9) into (6.14), it can be shown that

A 1 $$\begin{array}{rl} R_{1}(k) & =\int_{-\infty }^{\infty }\{\frac{\mu \bar{z_{0}}}{z_{0}^{2}}\left[\frac{\pi }{2\theta }\;\coth\left[\frac{\pi (\eta -\eta_{0})}{2\theta }\right]-\frac{\pi }{2\theta }\;\coth\left[\frac{\pi (\eta -\bar{\eta_{0}})}{2\theta }\right]\right]\\ & \;+\frac{\mu }{z_{0}}\left[1-\frac{\bar{z_{0}}}{z_{0}}\right]{\left(\frac{\pi }{2\theta }\right)}^{2}{\mathrm{cosech}}^{2}\left(\frac{\pi (\eta -\eta_{0})}{2\theta }\right)\}e^{-ik\eta }\;d\eta \end{array}$$

and

A 2 $$\begin{array}{rl} R_{2}(k) & =\int_{\infty +i\theta }^{-\infty +i\theta }\{\frac{\mu \bar{z_{0}}}{z_{0}^{2}}\left[\frac{\pi }{2\theta }\;\coth\left[\frac{\pi (\eta -\eta_{0})}{2\theta }\right]-\frac{\pi }{2\theta }\;\coth\left[\frac{\pi (\eta -\bar{\eta_{0}})}{2\theta }\right]\right]\\ & \;+\frac{\mu }{z_{0}}\left[e^{-2i\theta }-\frac{\bar{z_{0}}}{z_{0}}\right]{\left(\frac{\pi }{2\theta }\right)}^{2}{\mathrm{cosech}}^{2}\left(\frac{\pi (\eta -\eta_{0})}{2\theta }\right)\}e^{-ik\eta }\;d\eta . \end{array}$$

In the expression for *R*_2_(*k*), we have used the fact that $\bar{\eta }=\eta -2i\theta$ on side 2 and the 2*iθ*-periodicity of the coth function. Hence

A 3 $$\begin{array}{rl} R(k) & =R_{1}(k)+R_{2}(k)=\frac{\mu \bar{z_{0}}}{z_{0}^{2}}\oint_{\partial D}\left\{[\frac{\pi }{2\theta }\;\coth\left[\frac{\pi (\eta -\eta_{0})}{2\theta }\right]-\frac{\pi }{2\theta }\;\coth\left[\frac{\pi (\eta -\bar{\eta_{0}})}{2\theta }\right]\right]\\ & \;-{\left(\frac{\pi }{2\theta }\right)}^{2}{\mathrm{cosech}}^{2}\left(\frac{\pi (\eta -\eta_{0})}{2\theta }\right)\}e^{-ik\eta }\;d\eta +\frac{\mu }{z_{0}}\{\int_{-\infty }^{\infty }{\left(\frac{\pi }{2\theta }\right)}^{2}{\mathrm{cosech}}^{2}\left(\frac{\pi (\eta -\eta_{0})}{2\theta }\right)e^{-ik\eta }\;d\eta \\ & \;+\;e^{-2i\theta }\int_{\infty +i\theta }^{-\infty +i\theta }{\left(\frac{\pi }{2\theta }\right)}^{2}{\mathrm{cosech}}^{2}\left(\frac{\pi (\eta -\eta_{0})}{2\theta }\right)e^{-ik\eta }\;d\eta \}, \end{array}$$

where the closed contour ∂*D* is the boundary of the channel region in the *η*-plane shown in figure 1. Residue calculus can be used to show that

A 4 $$\begin{array}{rl} \frac{\pi }{2\theta }\int_{-\infty }^{\infty }\left[\coth\left[\frac{\pi (\eta -\eta_{0})}{2\theta }\right]-\coth\left[\frac{\pi (\eta -\bar{\eta_{0}})}{2\theta }\right]\right]e^{-ik\eta }\;d\eta & =\frac{2\pi i(e^{-ik\eta_{0}}-e^{-ik\bar{\eta_{0}}+2k\theta })}{1-e^{2k\theta }},\\ \int_{-\infty }^{\infty }{\left(\frac{\pi }{2\theta }\right)}^{2}{\mathrm{cosech}}^{2}\left(\frac{\pi (\eta -\eta_{0})}{2\theta }\right)e^{-ik\eta }\;d\eta & =\frac{2\pi \;e^{-ik\eta_{0}}}{1-e^{2k\theta }}\\ \;\mathrm{and}\;\int_{\infty +i\theta }^{-\infty +i\theta }{\left(\frac{\pi }{2\theta }\right)}^{2}{\mathrm{cosech}}^{2}\left(\frac{\pi (\eta -\eta_{0})}{2\theta }\right)e^{-ik\eta }\;d\eta & =\frac{2\pi k\;e^{-ik\eta_{0}}}{1-e^{-2k\theta }}. \end{array}\}$$

These results are all derived by considering integrals of the integrands around the contour bounding the infinite channel region 0<Im[*η*]<2*θ* and noticing that, owing to the 2*iθ*-periodicity of the coth and *cosech*^2^ functions, the contribution from the edge along Im[*η*]=2*θ* is a multiple of that coming from the edge along Im[*η*]=0. Moreover, all integrands are meromorphic in the channel region and decay as |η|→∞ so the sum of the two integrals contributed by the upper and lower boundaries of the channel, which is a multiple of the integral we seek to compute, equals the sum of residue contributions from the enclosed poles. Consequently, we find

A 5 $$\begin{array}{rl} R_{1}(k) & =\frac{\mu \bar{z_{0}}}{z_{0}^{2}}\left[\frac{2\pi i(e^{-ik\eta_{0}}-e^{-ik\bar{\eta_{0}}+2k\theta })}{1-e^{2k\theta }}\right]+\frac{\mu }{z_{0}}\left[1-\frac{\bar{z_{0}}}{z_{0}}\right]\frac{2\pi ke^{-ik\eta_{0}}}{1-e^{2k\theta }},\\ & =\frac{2\pi i\mu (1+ik)}{1-e^{2k\theta }}\bar{z_{0}}z_{0}^{-ik-2}+\frac{2\pi i\mu }{1-e^{-2k\theta }}{\bar{z_{0}}}^{1-ik}z_{0}^{-2}+\frac{2\pi k\mu }{1-e^{2k\theta }}z_{0}^{-ik-1}\\ \;\mathrm{and}\;\;R(k) & =\frac{\mu \bar{z_{0}}}{z_{0}^{2}}\left[2\pi ie^{-ik\eta_{0}}-2\pi k\;e^{-ik\eta_{0}}\right]+\frac{\mu }{z_{0}}\left[\frac{2\pi k\;e^{-ik\eta_{0}}}{1-e^{2k\theta }}+e^{-2i\theta }\;\frac{2\pi k\;e^{-ik\eta_{0}}}{1-e^{-2k\theta }}\right]\\ & =2\pi i\mu (1+ik)\bar{z_{0}}z_{0}^{-ik-2}+2\pi k\mu \left(\frac{1-e^{2\theta (k-i)}}{1-e^{2k\theta }}\right)z_{0}^{-ik-1}. \end{array}\}$$

## Appendix B. Semi-infinite wall: *θ*=2*π*

This appendix derives the solution for a stresslet exterior to a semi-infinite wall using a conformal mapping method that is quite distinct from the transform approach presented in the main body of the paper. This is the case *θ*=2*π*. It has also been considered in [13] using similar methods to those used below. This conformal mapping approach to finding solutions for stresslets near walls with corners of angle 2*π* was first implemented (in a non-wedge geometry) by Crowdy & Samson [14].

Consider the conformal mapping from the unit disc to the region exterior to a semi-infinite flat wall. It is given by

B 1 $$z=-{\left(\frac{1-\zeta }{1+\zeta }\right)}^{2}.$$

We now show that, in terms of the parametric *ζ* variable, the exact solution for this problem is

B 2 $$F(\zeta )=\frac{a}{\zeta -\zeta_{0}}+\frac{\bar{b}}{1/\zeta -\bar{\zeta_{0}}}+\frac{\bar{c}}{{\left(1/\zeta -\bar{\zeta_{0}}\right)}^{2}}$$

for some complex *a*,*b* and *c* to be determined. The no-slip boundary condition on |*ζ*|=1 implies

B 3 $$u-iv=-\bar{F(\zeta )}+\bar{z(\zeta )}\frac{F^{\prime }(\zeta )}{z^{\prime }(\zeta )}+G(\zeta )=0.$$

Since $\bar{z}=z$ on |*ζ*|=1 where $\bar{\zeta }=1/\zeta$ then

B 4 $$G(\zeta )=\bar{F}(1/\zeta )-\frac{z(\zeta )}{z^{\prime }(\zeta )}F^{\prime }(\zeta ).$$

From (B 2), we easily find

B 5 $$\begin{array}{rl} F^{\prime }(\zeta ) & =-\frac{a}{{\left(\zeta -\zeta_{0}\right)}^{2}}+\frac{\bar{b}}{\zeta^{2}}\frac{1}{{\left(1/\zeta -\bar{\zeta_{0}}\right)}^{2}}+2\frac{\bar{c}}{\zeta^{2}}\frac{1}{{\left(1/\zeta -\bar{\zeta_{0}}\right)}^{3}}\\ \;\mathrm{and}\;\bar{F}(1/\zeta ) & =\frac{\bar{a}}{1/\zeta -\bar{\zeta_{0}}}+\frac{b}{\zeta -\zeta_{0}}+\frac{c}{{\left(\zeta -\zeta_{0}\right)}^{2}}. \end{array}\}$$

It follows from a Taylor expansion, i.e.

B 6 $$z-z_{0}=z^{\prime }(\zeta_{0})(\zeta -\zeta_{0})+\frac{z^{\prime \prime }(\zeta_{0})}{2!}{\left(\zeta -\zeta_{0}\right)}^{2}+\frac{z^{\prime \prime \prime }(\zeta_{0})}{3!}{\left(\zeta -\zeta_{0}\right)}^{3}\ldots$$

that near *ζ*_0_,

B 7 $$\frac{1}{\zeta -\zeta_{0}}=\frac{z^{\prime }(\zeta_{0})}{z-z_{0}}\left[1+\frac{z^{\prime \prime }(\zeta_{0})}{2z^{\prime }(\zeta_{0})}(\zeta -\zeta_{0})+\frac{z^{\prime \prime \prime }(\zeta_{0})}{3!z^{\prime }(\zeta_{0})}{\left(\zeta -\zeta_{0}\right)}^{2}\ldots \right].$$

But we also have the Taylor expansion

B 8 $$\zeta -\zeta_{0}=\zeta^{\prime }(z_{0})(z-z_{0})+\frac{\zeta^{\prime \prime }(z_{0})}{2!}{\left(z-z_{0}\right)}^{2}+\cdots ,$$

which can be used, on substitution into (B 7), to establish that

B 9 $$\frac{1}{\zeta -\zeta_{0}}=\frac{z^{\prime }(\zeta_{0})}{z-z_{0}}+\frac{z^{\prime \prime }(\zeta_{0})}{2z^{\prime }(\zeta_{0})}+(z-z_{0})\left[\frac{z^{\prime \prime }(\zeta_{0})\zeta^{\prime \prime }(z_{0})}{4}+\frac{z^{\prime \prime \prime }(\zeta_{0})\zeta^{\prime }{\left(z_{0}\right)}^{2}}{6}\right]+\cdots$$

or, on use of the identities,

B 10 $$\zeta^{\prime }(z)=\frac{1}{z^{\prime }(\zeta )}\;\mathrm{and}\;\zeta^{\prime \prime }(z)=-\frac{z^{\prime \prime }(\zeta )}{z^{\prime }{\left(\zeta \right)}^{3}}$$

that

B 11 $$\frac{1}{\zeta -\zeta_{0}}=\frac{z^{\prime }(\zeta_{0})}{z-z_{0}}+\frac{z^{\prime \prime }(\zeta_{0})}{2z^{\prime }(\zeta_{0})}+\frac{\left(z-z_{0}\right)}{z^{\prime }(\zeta_{0})}\left[-\frac{1}{4}{\left[\frac{z^{\prime \prime }(\zeta_{0})}{z^{\prime }(\zeta_{0})}\right]}^{2}+\frac{1}{6}\frac{z^{\prime \prime \prime }(\zeta_{0})}{z^{\prime }(\zeta_{0})}\right]+\cdots .$$

Now from (B 1) we find

B 12 $$\frac{z^{\prime }(\zeta )}{z(\zeta )}=-\frac{4}{1-\zeta^{2}},$$

implying, with two successive differentiations, that

B 13 $$\begin{array}{rl} \frac{z^{\prime \prime }(\zeta )}{z^{\prime }(\zeta )} & =-\frac{4}{1-\zeta^{2}}-\frac{8\zeta }{{\left(1-\zeta^{2}\right)}^{2}}\frac{z}{z^{\prime }}=\frac{2(\zeta -2)}{\left(1-\zeta^{2}\right)}\\ \;\mathrm{and}\;\frac{z^{\prime \prime \prime }(\zeta )}{z^{\prime }(\zeta )} & ={\left[\frac{z^{\prime \prime }(\zeta )}{z^{\prime }(\zeta )}\right]}^{2}+\frac{2(1-4\zeta +\zeta^{2})}{{\left(1-\zeta^{2}\right)}^{2}}. \end{array}\}$$

Hence, near *ζ*_0_,

B 14 $$\frac{1}{\zeta -\zeta_{0}}=\frac{A}{z-z_{0}}+B+C(z-z_{0})+O{\left(z-z_{0}\right)}^{2},$$

where

B 15 $$A=\frac{4(1-\zeta_{0})}{{\left(1+\zeta_{0}\right)}^{3}},\;B=\frac{\zeta_{0}-2}{1-\zeta_{0}^{2}}\;\mathrm{and}\;C=-\frac{\left(1+\zeta_{0}\right)}{4{\left(1-\zeta_{0}\right)}^{3}}.$$

First, substitution of (B 14) into (B 2) we find that, near *ζ*_0_,

B 16 $$F(\zeta )=\frac{a}{\zeta -\zeta_{0}}+Q+R(\zeta -\zeta_{0})+\cdots ,$$

where

B 17 $$Q=\frac{\bar{b}}{1/\zeta_{0}-\bar{\zeta_{0}}}+\frac{\bar{c}}{{\left(1/\zeta_{0}-\bar{\zeta_{0}}\right)}^{2}}\;\mathrm{and}\;R=\frac{\bar{b}}{\zeta_{0}^{2}}\frac{1}{{\left(1/\zeta_{0}-\bar{\zeta_{0}}\right)}^{2}}+\frac{2\bar{c}}{\zeta_{0}^{2}}\frac{1}{{\left(1/\zeta_{0}-\bar{\zeta_{0}}\right)}^{3}}.$$

Hence, as a function of *z*,

B 18 $$\begin{array}{rl} F(\zeta ) & =a\left[\frac{A}{z-z_{0}}+B+C(z-z_{0})+O{\left(z-z_{0}\right)}^{2}\right]+Q+\frac{R}{z^{\prime }(\zeta_{0})}(z-z_{0})+\cdots \\ & =\frac{aA}{z-z_{0}}+(aB+Q)+\left[aC+\frac{R}{z^{\prime }(\zeta_{0})}\right](z-z_{0})+\cdots , \end{array}$$

implying the condition

B 19 aA=μ,

which determines *a* in order that, near *z*_0_,

B 20 $$f(z)=\frac{\mu }{z-z_{0}}+f_{0}+f_{1}(z-z_{0})+\cdots$$

with

B 21 $$f_{0}=aB+Q\;\mathrm{and}\;f_{1}=aC+\frac{R}{z^{\prime }(\zeta_{0})}.$$

Next, on substitution of (B 20) and (B 14) into (B 4) we find

B 22 $$\begin{array}{rl} G(\zeta ) & =\bar{F}(1/\zeta )-zf^{\prime }(z)\\ & =\frac{c}{{\left(\zeta -\zeta_{0}\right)}^{2}}+\frac{b}{\left(\zeta -\zeta_{0}\right)}+\frac{\bar{a}}{1/\zeta -\bar{\zeta_{0}}}-z\left[-\frac{\mu }{{\left(z-z_{0}\right)}^{2}}+f_{1}+\cdots \right]\\ & =c\left[\frac{A^{2}}{{\left(z-z_{0}\right)}^{2}}+\frac{2AB}{\left(z-z_{0}\right)}+(B^{2}+2AC)+\cdots \right]+b\left[\frac{A}{\left(z-z_{0}\right)}+B+\cdots \right]\\ & \;+\frac{\bar{a}}{1/\zeta_{0}-\bar{\zeta_{0}}}+\frac{\mu z_{0}}{{\left(z-z_{0}\right)}^{2}}+\frac{\mu }{z-z_{0}}-f_{1}z_{0}+O(z-z_{0}), \end{array}$$

which gives the local behaviour of *G*(*ζ*) as a function of *z* (rather than *ζ*). As a result we now see that we require

B 23 $$cA^{2}+\mu z_{0}=\mu \bar{z_{0}},\;2ABc+bA+\mu =0,$$

which are two equations determining both *c* and *b* in order that

B 24 $$G(\zeta )=\frac{\mu \bar{z_{0}}}{{\left(z-z_{0}\right)}^{2}}+g_{0}+O(z-z_{0}),$$

with

B 25 $$g_{0}=c(B^{2}+2AC)+bB-f_{1}z_{0}+\frac{\bar{a}}{1/\zeta_{0}-\bar{\zeta_{0}}}.$$
